# Phytochemical and biological evaluation of methanolic extracts as a preventive measure for antioxidant and Anticoccidial Eimeria columbae Oocysts

**DOI:** 10.1371/journal.pone.0353429

**Published:** 2026-07-10

**Authors:** Mutee Murshed, Jameel Al-Tamimi, Khalid Elfaki Ibrahim, Saleh Al-Quraishy

**Affiliations:** Department of Zoology, College of Science, King Saud University, Riyadh, Saudi Arabia; Tanta University Faculty of Agriculture, EGYPT

## Abstract

**Background:**

Herbal products are rich in the source of bioactive ingredients, including phenols, flavonoids, tannins, and essential oil components such as cinnamaldehyde, eugenol, and 1,8-cineole, and have significant potential for therapeutic applications that are associated with antioxidant and anticoccidial activities.

**Aims:**

This study aimed to investigate the phytochemical quantification and antioxidant anticoccidial assay in methanolic extracts of *Cinnamomum verum* and *Laurus nobilis*.

**Methods:**

Total phenol, flavonoid, and tannin content was measured using standard spectrophotometric methods. Phytochemical properties were characterized using Fourier transform infrared spectroscopy (FTIR). Antioxidant activity was assessed using 2,2-diphenyl-1-picrylhydrazyl (DPPH) and (2,2-diphenyl-1-picrylhydrazyl (ABTS) free radical scavenging assays. In vitro anticoccidial activity was assessed using seven concentrations*:* 10, 25, 50, 100, 150, and 200 mg/mL of *C. verum and L. nobilis*, in addition to K₂Cr₂O₇ as the control at 24, 48, 72, and 96 hours.

**Results:**

FTIR analysis revealed characteristic absorption bands in the *C. verum* and *L. nobilis* extracts, indicating the presence of diverse functional groups associated with phenolic and related phytochemical constituents, as evidenced by their characteristic absorption bands across the 400–4000 cm ⁻ ¹ range. All extracts contained measurable levels of phenols, flavonoids, and tannins. The highest total phenolic content was recorded in *C. verum* (63.56 ± 0.552 mg/g dry weight) and *L.* *nobilis* (57.40 ± 6.05 mg/g dry weight). All extracts exhibited strong antioxidant activity, with IC₅₀ values for DPPH (12.59 ± 0.51 and 30.32 ± 3.22 μg/mL) and ABTS (13.95 ± 0.35, 19.56 ± 4.89 μg/mL) assays to *C. verum and L. nobilis*. The extracts exhibited dose- and time-dependent inhibition of *E. columbae* sporulated oocysts, at 200 mg/ml, 75.67% at *C. verum* and 72.57% at *L. nobilis* (p < 0.05) at higher concentrations at 96 h, alongside increased oocyst destruction of ~12%.

**Conclusion:**

The findings demonstrated that the extracts of *C. verum and L. nobilis* leaves contain bioactive phytochemicals that inhibit coccidia oocysts in vitro, suggesting a potentially effective and safe natural option for managing coccidiosis in Domestic Pigeons.

## Introduction

The increasing interest in natural plant products for disease prevention and treatment is largely attributed to their lower cost and reduced side effects compared with synthetic drugs [1]. Medicinal plants remain an important source of bioactive compounds and continue to influence modern pharmacological research because many of their metabolites exhibit antioxidant, antimicrobial, and antiparasitic properties [2,3]. Phenolic compounds, flavonoids, tannins, and volatile constituents can act as hydrogen- or electron-donating molecules, stabilize free radicals, chelate pro-oxidant metals, and interfere with essential cellular targets, thereby contributing to both antioxidant and anti-infective effects [4].

Cinnamomum verum was selected because previous studies have reported its antiparasitic and antimicrobial potential [5]. Its essential oil contains bioactive constituents such as cinnamaldehyde, cinnamyl acetate, and eugenol, which can alter membrane permeability, disrupt energy metabolism, inhibit key enzymes, and induce redox imbalance in susceptible parasites and microbes [6]. Cinnamon extracts and oils have also shown activity against several parasites, including Leishmania, Plasmodium, and gastrointestinal helminths, supporting their value as candidates for antiparasitic investigation [7]. *Laurus nobilis* has biological activity against a range of infectious agents and its antiparasitic effects [8]. The essential oil of *L. nobilis*, characterized by compounds such as 1,8-cineole, linalool, eugenol, and methyl eugenol, has demonstrated antiprotozoal, helminthic, and insecticidal activity in previous studies [9]. The traditional medicinal use of *L. nobilis* in treating gastrointestinal disorders, diarrhea, and inflammations is consistent with its potential antiparasitic properties. Furthermore, experimental reports have shown that *L. nobilis* extracts can impair the survival and motility of parasites [8].

In poultry, coccidial infection caused by Eimeria tenella is a major health problem because the parasite completes part of its life cycle through the formation and sporulation of oocysts in the external environment [10]. After being shed in feces, unsporulated oocysts undergo sporulation under suitable temperature, moisture, and oxygen conditions, producing infective sporulated oocysts that can initiate new infections [11]. This process is critical for the transmission of coccidiosis, and any factor that delays, suppresses, or disrupts sporulation can reduce parasite infectivity and disease spread [12]. Recent studies have shown that adding plant-based antioxidants to feed significantly improves the physiological performance and health status of poultry [13]. Phytochemicals, such as phenolic compounds, flavonoids, and essential oil components, mitigate oxidative stress through enzymes like superoxide dismutase, catalase, and glutathione peroxidase [14]. In animals, oxidative stress is closely linked to impaired growth, immune dysfunction, intestinal damage. Adding antioxidant-rich plant extracts has been shown to improve feed efficiency, growth rate, immune response, intestinal structure, and resistance to infections and parasites, particularly under conditions of environmental and metabolic stress [15]. Despite this progress, there is still insufficient information on the antioxidant activities, such as those tested using ABTS and DPPH assays, and to assess their anticoccidial activity against *Eimeria columbae* oocysts in comparison with amprolium.

Therefore, this study was designed to comparatively evaluate the methanolic extracts of *C. verum* and *L. nobilis* for their phytochemical composition, antioxidant potential, and anticoccidial activity against *E. columbae* oocysts. In particular, we aimed to quantify total phenolics, flavonoids, and tannins; assess radical-scavenging activity using ABTS and DPPH assays; and determine the ability of both extracts to inhibit oocyst sporulation in vitro. To the best of our knowledge, this is the first study to compare these two plant species under the same experimental framework for both antioxidant and anticoccidial evaluation, thereby providing a clearer basis for their potential use as natural alternatives in coccidiosis control.

## Materials and methods

### Ethics approval

The oocysts that infect pigeons were isolated from the feces of naturally infected pigeons, and the pigeons were not sacrificed. The effect of the extracts was tested as a preventive procedure to prevent the oocysts from sporulating, because if the oocysts sporulate, they will cause infection and the pigeons’ infection.

### Plant material collection and botanical authentication

The herbaceous plants *Cinnamomum verum (C. verum) and Laurus nobilis (L. nobilis)* were purchased from local spice markets in Riyadh, Saudi Arabia. Their botanical identities were verified by Prof. Mohamed El-Sheikh and Dr. Rajakrishnan R. from the Department of Botany, King Saud University, and voucher specimens were deposited under accession numbers KSU No: 10504 and 24649, respectively.

### Preparation of plant materials and extracts

The bark of *C. verum* and the leaves of *L. nobilis* were cleaned and shade-dried at 25 °C for ten days. The dried materials were ground into fine powder and stored in sealed plastic bags at 4 °C until further use. For extraction, 150 g of powdered plant material was macerated in 70% methanol and placed on a shaker at 4 °C for 24 h. The mixture was filtered through Whatman No. 1 filter paper, and the combined filtrates were concentrated under reduced pressure using a Büchi R 300 rotary evaporator (water bath 50 °C, 120 rpm, ~ 80 mbar), following the method of Yang et al. (2016), [16]. The extraction yield ranged between 40–50 g. The crude extracts were dissolved in distilled water for subsequent analyses.

### FT IR Analysis of *C. verum* and *L. nobilis* Extracts

Fourier-transform infrared (FT IR) spectroscopy was used to identify the major functional groups in the extracts. Each sample was ground and homogenized with potassium bromide (KBr) powder in a 1:99 w/w ratio, pressed into pellets, and analyzed using a Thermo Scientific NICOLET 6700 FT IR spectrometer at 25 °C with a resolution of 4 cm ⁻ ¹, as described by Altemimi et al. (2017) [17].

### Determination of total phenolic content

The Folin–Ciocalteu colorimetric method measured the extracts’ total phenolic content was done according to Al-Quraishy et al. (2026) [18]. In brief, 100 μL of sample or gallic acid standard was combined with 200 μL of 10% Folin-Ciocalteu reagent in 2 mL Eppendorf tubes. The mixture was incubated in the dark for 2 hours after adding 800 μL of 0.7 M Na₂CO₃ after brief vortexing. Using 765 nm absorbance, TPC was calculated as mg gallic acid equivalents (GAE/g DW).

### Determination of total flavonoid content

Total flavonoids were quantified using the aluminum chloride colorimetric assay was done according to Al-Quraishy et al. (2022) [18]. Samples (30 μL) were mixed with 160 μL methanol, followed by 30 μL of 10% AlCl₃ in methanol, 850 μL distilled water, and 30 μL of 1 M sodium acetate. After vortexing, mixtures were left at room temperature for 30 min, and absorbance was recorded at 415 nm. Results were expressed as mg quercetin equivalents (QE/g DW).

### Determination of total tannin content

The total tannin content (TTC) was determined following the modified Folin–Ciocalteu method [18]. In brief, 0.1 mL of the sample extract was mixed with 1.5 mL Milli Q water and 1 mL diluted Folin–Ciocalteu reagent. After adding 0.8 mL of 7.5% NaHCO₃, the mixture was incubated at 45 °C for 45 min in the dark. Absorbance was measured at 700 nm, and results were expressed as mg tannic acid equivalents (TAE/g DW.

### ABTS radical scavenging assay

The antioxidant capacity was evaluated using the ABTS radical cation decolorization assay, as described by Murshed et al. (2025) [19]. ABTS⁺ radicals were generated by mixing 7 mM ABTS with 2.45 mM potassium persulfate and incubating the mixture in the dark for 16 h at 25 °C. The working solution was diluted with methanol to an absorbance of 0.70 ± 0.02 at 734 nm. Equal volumes (1 mL) of the ABTS⁺ solution and sample were mixed, incubated for 7 min, and absorbance was measured at 734 nm. Trolox was used as a standard, and antioxidant activity was expressed as µM Trolox equivalent antioxidant capacity (TEAC).

### DPPH radical scavenging assay

The free radical scavenging ability of the extracts was determined using the DPPH assay, following Desmarchelier et al. (1997) [20]. A 0.1 mM DPPH methanolic solution was mixed with various concentrations of the extract (12.5–150 μg/mL) in a 2.4 mL:1.6 mL ratio. After vertexing, the samples were incubated in the dark at room temperature for 30 min, and absorbance was recorded at 517 nm. The percentage inhibition of DPPH radicals was calculated as:


$$\begin{array}{c} \%\text{DPPH radical scavenging activity}={\text{A}}_{\text{0}}\left({\text{A}}_{\text{0}}-{\text{A}}_{\text{1}}\right)\;\times \text{ 100} \end{array}$$

where A0 represents absorbance of the control and A1 is that of the extract/standard. The percentage inhibition versus concentration graph allowed for IC_50_ calculation; experiments were repeated three times at each concentration.

## Antiparasitic activity

### Assay of sporulation inhibition

Commonly infected wild pigeons were used to isolate the parasite. Feces-derived non-sporulated oocysts were sporulated in 2.5% potassium dichromate. The assay used 24-well flat-bottom microtitration plates [21]. Two-fold serial dilution was performed on methanolic extracts of *C. verum* and *L. nobilis* at 10, 25, 50, 200, 150, and 200 mg/mL. VAPCO compound amprolium (Amman, Jordan) served as the positive control, while K₂Cr₂O₇ served as the negative control. In each well, 1 × 10³ *E. columbae* oocysts were dissolved in 2 mL of K₂Cr₂O₇. Plates were incubated at 28°C with 60–80% humidity consistently. The experiment employed three samples per treatment. All plates were semi-covered and shook regularly. sporulated and non-sporulated oocysts were observed at 24, 48, 72, and 96 h [21]. A light microscope (BX51TF, OLYMPUS, Tokyo, Japan) was used to inspect 5 µL of solution at 40x magnification. Each group was examined. The percentages of oocysts were computed utilizing the formulas:


$$\begin{array}{c} \text{Sporulation }(\%)\;=\text{ Sporulated oocysts}/\text{ Total oocysts }\times \text{100}. \end{array}$$

$$\begin{array}{c} \text{ Inhibition}\%\;=\text{ Sporulation of control }-\text{ sporulation of medicine}/\text{Sporulation of control }\times \text{100} \end{array}$$

### Statistical analysis

All experiments were conducted in triplicate, and results are presented as mean ± standard deviation. Data were analyzed using factorial ANOVA to examine the effects of plant species, concentration, and time, followed by Tukey’s post-hoc multiple comparison test to determine significant differences among groups. Statistical significance was set at *P* < 0.05 [22].

## Results

**Fig 1:**
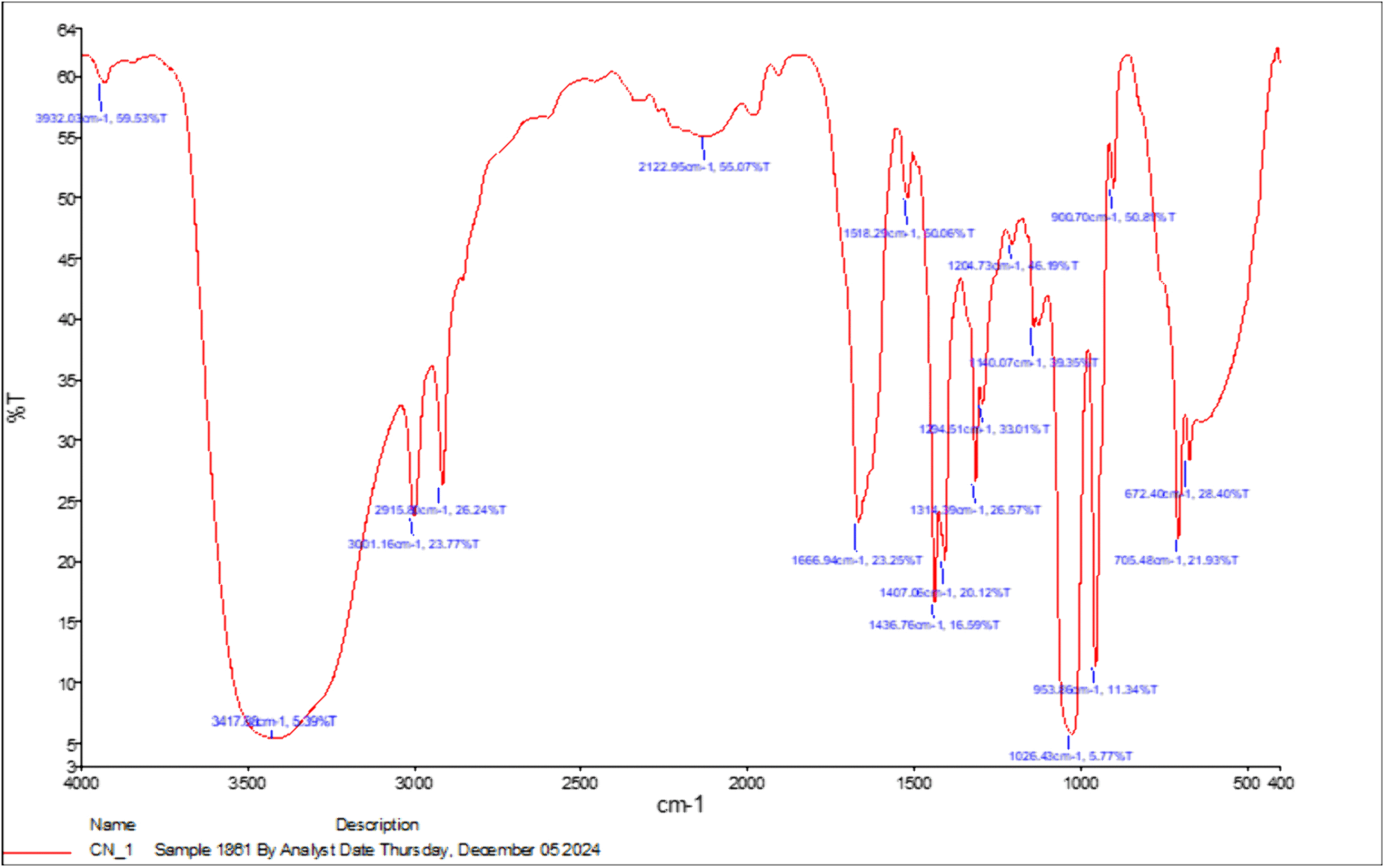
FTIR Chromatogram of methanolic extract of *C. verum* extracts.

Fig 1 displays the transmittance spectrum of *C. verum* extract across ~400–4000 cm ⁻ ¹, with peaks corresponding to functional group/absorption band descriptions of moieties as tabulated. Key features include broad O-H stretches at 3932 cm ⁻ ¹ (59.53% T; alcohols) and 3417 cm ⁻ ¹ (5.33% T; primary amines), indicative of hydrogen-bonded phenolics; strong N-H at 2915 cm ⁻ ¹ (26.24% T; amine salts); conjugated C = O at 1666 cm ⁻ ¹ (23.25% T; ketones, e.g., cinnamaldehyde); phenolic O-H bend at 1314 cm ⁻ ¹ (26.57% T); and clustered C-O stretches (1294–1026 cm ⁻ ¹; aromatic esters/ethers/alcohols, low T ~ 5–46%) signaling flavonoids/polyols. Aromatic C-H bends (672–900 cm ⁻ ¹) confirm benzene derivatives. Collectively, these 18 + bands highlight C. verum’s polyphenolic richness, consistent with its known essential oil profile (Table 1).

**Table 1:** Fourier spectrum of *C. verum* bark extracts by frequency range.

Absorption cm ^− 1^	Appearance	Transmittance %	Groups	Compound Class
3932.03	Medium	59.53	O-H stretching	alcohol
3417.88	Medium	5.33	N-H stretching	primary amine
3001.16	weak, broad	23.77	O-H stretching	alcohol
2915.8	strong, broad	26.24	N-H stretching	amine salt
2122.95	strong	55.07	N = N = N stretching	azide
1666.94	strong	23.25	C = O stretching	conjugated etone
1518.29	strong	50.06	N-O stretching	nitro compound
1436.76	medium	16.59	C-H bending	alkane
1407.06	medium	20.12	O-H bending	carboxylic acid
1314.39	medium	26.57	O-H bending	phenol
1294.51	strong	33.01	C-O stretching	aromatic ester
1204.73	strong	46.19	C-O stretching	alkyl aryl ether
1140.07	strong	39.35	C-O stretching	tertiary alcohol
1026.43	strong	5.77	C-O stretching	secondary alcohol
953.86	strong	11.34	C = C bending	alkene
900.7	strong	50.81	C-H bending	1,2,4 risubstituted
705.84	strong	21.93	C-H bending	benzene erivative
672.4	strong	28.4	C-H bending	benzene

**Fig 2:**
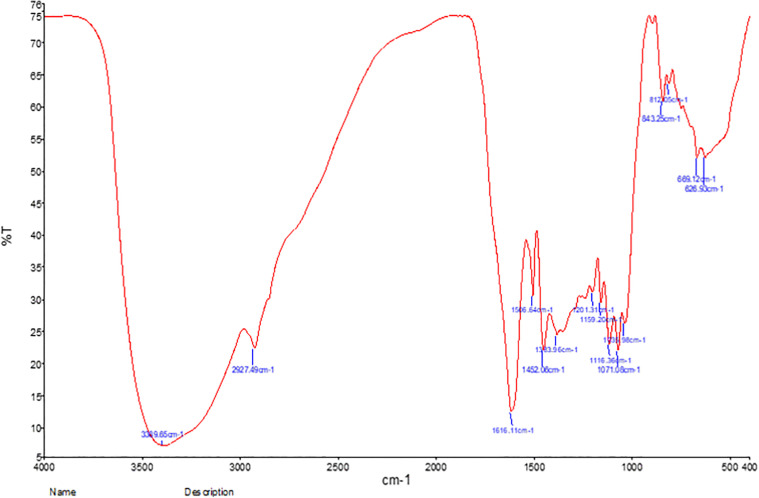
FT-IR spectrum of *L. nobilis* extracts based on the frequency range.

Fig 2 presents *L. nobilis* spectrum with fewer, moderately intense peaks, detailed in Table 2. Dominant signals are O-H stretches at 3409 cm ⁻ ¹ (21% T; sharp, alcohols) and 2927 cm ⁻ ¹ (29% T; broad); strong C = O at 1712 cm ⁻ ¹ (36% T; aliphatic ketones); C = C at 1606 cm ⁻ ¹ (24% T); carboxylic O-H at 1368 cm ⁻ ¹ (38% T); and C-O stretches (1152–1066 cm ⁻ ¹; ethers/alcohols, 26–32% T). Unique features include S = O at 1033 cm ⁻ ¹ (23% T; sulfoxides) and halo C-Cl at 596 cm ⁻ ¹ (63% T), (Table 2).

**Table 2:** FT-IR spectra analysis of dried *L. nobilis* plant leaf extracts.

Absorption cm ^− 1^	Peak Details	Transmittance (%)	Functional Group	Compound class
3409.71	medium, sharp	21	O-H stretching	alcohol
2927.1	weak, broad	29	O-H stretching	alcohol
1712.53	Strong	36	C = O stretching	aliphatic ketone
1606.44	medium	24	C = C stretching	aliphatic ketone
1515.18	strong	31	N-O stretching	nitro compound
1450.96	medium	28	C-H bending	alkane
1368.61	strong	38	O-H bending	carboxylic acid
1270.36	strong	37	C-N stretching	aromatic amine
1152.72	strong	32	C-O stretching	aliphatic ether
1122.52	strong	26	C-O stretching	secondary alcohol
1066.69	strong	28	C-O stretching	primary alcohol
1033.4	strong	23	S = O stretching	sulfoxide
818.35	medium	54	C = C bending	alkene
795.06	medium	58	C-H bending	1,4-disubstituted
596.17	strong	63	C-Cl stretching	halo compound

Compared to *C. verum, L. nobilis* shows simpler profiles skewed toward terpenoids/ketones rather than conjugated aromatics. Also, *C. verum* (Fig. 1/Tab. 1) exhibits ~20% more peaks with lower transmittance (stronger absorptions) in bioactive zones (1200–1700 cm ⁻ ¹), signaling higher metabolite density versus *L. nobilis’* moderate ketone/ether focus (Fig. 2/Tab. 2). This aligns with cinnamon’s superior antioxidant potential, as conjugated systems enhance radical stability. These spectra validate the extracts’ phytochemical integrity for downstream bioassays.

### Phytochemical quantification

**Fig 3:**
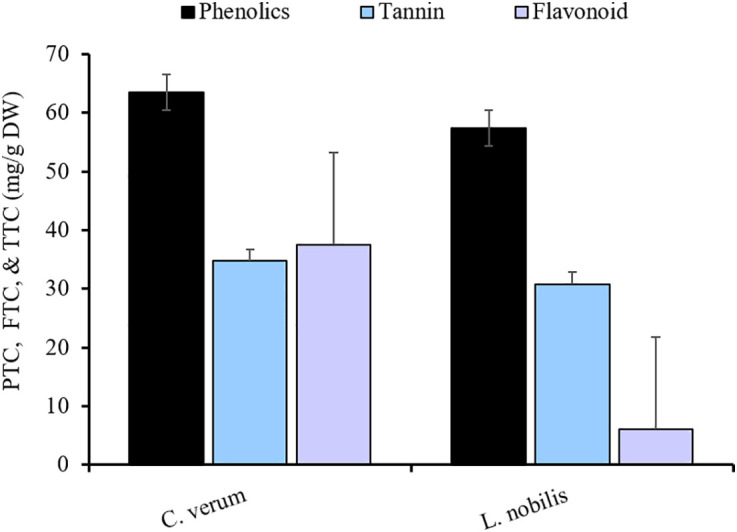
TPC, TTC, and TFC, of two therapeutic plant preparations.

Derived from the curves, *C. verum* exhibits ~10–13% higher TPC/TTC and 6 × higher TFC than *L. nobilis,* indicating superior polyphenol recovery and potential for antioxidant applications. These values reflect methodological precision (low SDs) and methanol’s efficacy (Fig 3).

**Fig 4:**
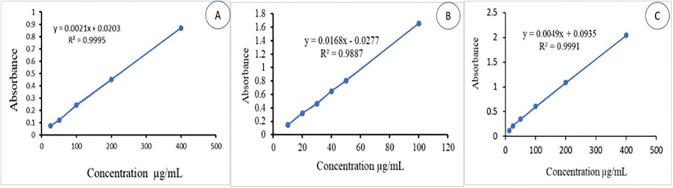
Standard calibration curves for the quantification of total phenolic content using gallic acid (A), total flavonoid content using chrysin (B), and total tannin content using tannic acid (C).

Fig 4AC depict the standard calibration curves used for quantitative phytochemical analysis via spectrophotometry, demonstrating high linearity essential for accurate determination of total phenolic, flavonoid, and tannin contents in the extracts. Figure 4A (Phenolics, Gallic Acid): A linear regression (y = 0.043x + 0.027; R² = 0.9897) plots absorbance at 765 nm against concentration (0–100 µg/mL), confirming the Folin-Ciocalteu assay’s precision for TPC interpolation (e.g., yielding 63.56 mg CE/g DW for *C. verum*). Figure 4B (Flavonoids, Chrysin): Strong linearity (y = 0.006x + 0.057; R² = 0.987) at 415 nm supports AlCl₃-based TFC quantification, enabling reliable values like 37.56 mg CE/g DW in *C. verum*. Figure 4C (Tannins, Tannic Acid): Excellent fit (y = 0.008x + 0.051; R² = 0.9901) at ~700 nm validates modified Folin-Ciocalteu for TTC (e.g., 34.81 mg GAE/g DW in *C. verum*).

The image illustrates these curves’ robust R² (>0.98), underscoring methodological reliability for extract analysis.

**Fig 5:**
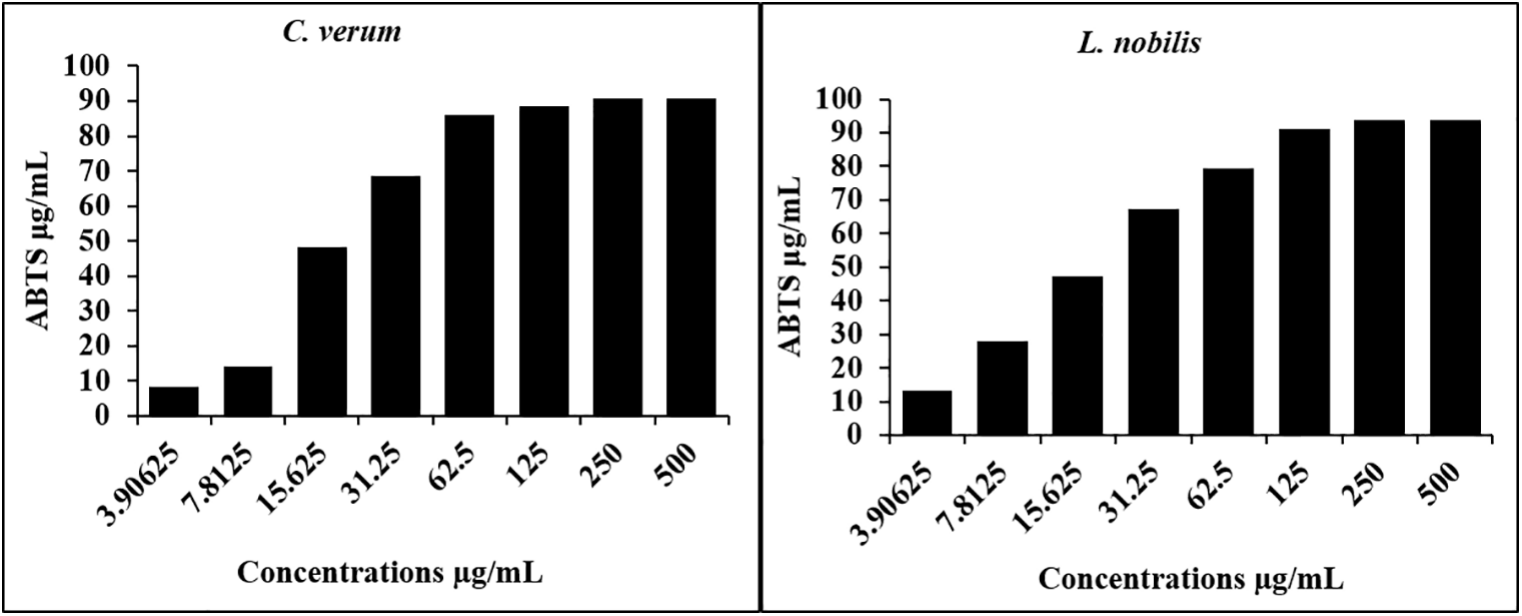
Evaluation of ABTS in *Cinnamomum verum* and *Laurus nobilis* extracts. The significances are the average of three tests, and they are expressed mg GAE/L.

Fig 5 showed that *C. verum* extract exhibited a concentration-dependent increase in ABTS radical scavenging activity, with inhibition rising from about 20% at 12.5 µg/mL to more than 90% at 150 µg/mL, indicating strong dose-responsive decolorization of ABTS- + radicals. In contrast, *L. nobilis* extract showed a similar trend, but with a slightly shallower slope, reaching approximately 85% inhibition at 150 µg/mL, which suggested comparatively lower radical-quenching efficiency.

**Fig 6:**
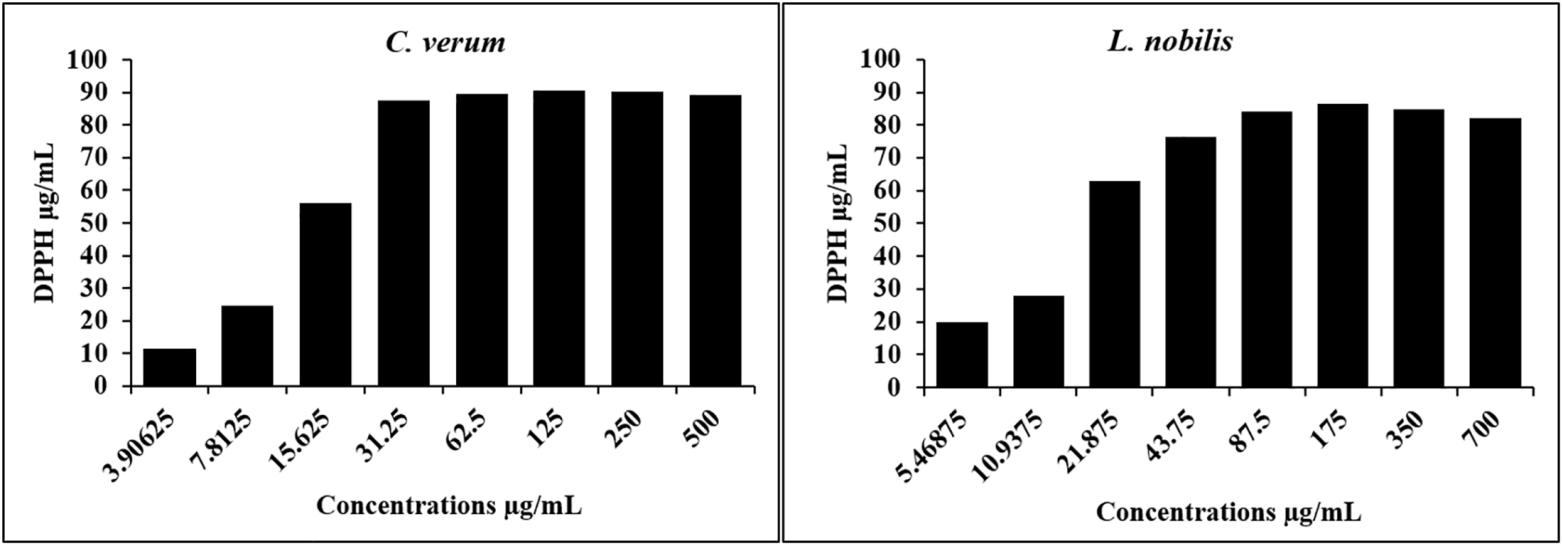
Evaluation of DPPH in *C. verum and L. nobilis* extracts. The significances are the average of three tests, and they are expressed mg GAE/L.

Fig 6 (*C. verum* DPPH): Strong scavenging from ~25% (12.5 µg/mL) to >95% (150 µg/mL) at 517 nm, characteristic of hydrogen atom transfer by polyphenols to DPPH-. Figure 8 (*L. nobilis* DPPH): Moderate activity (~15–80% across range), reflecting reduced capacity versus *C. verum* due to lower TFC/TTC.

The composite image confirms both extracts’ dose-response patterns, with *C. verum* consistently achieving higher % inhibition.

*C. verum* required 29–58% lower concentrations for 50% inhibition, confirming ~2–3 × greater antioxidant potency across both assays. These IC₅₀ correlate directly with its 6 × higher TFC (37.56 vs 6.09 mg QE/g), validating structure-activity relationships where flavonoids drive ROS neutralization (Table 3).

**Table 3:** IC₅₀ values (μg/mL) of DPPH and ABTS radical scavenging assays for *Cinnamomum verum* and *Laurus nobilis* extracts.

Extracts	IC50 (μg/mL)	
	DPPH	ABTS
*C. verum*	13.95 ± 0.35	12.59 ± 0.51
*L. nobilis*	19.56 ± 4.89	30.32 ± 3.22

### Antiparasitic activity

**Fig 7:**
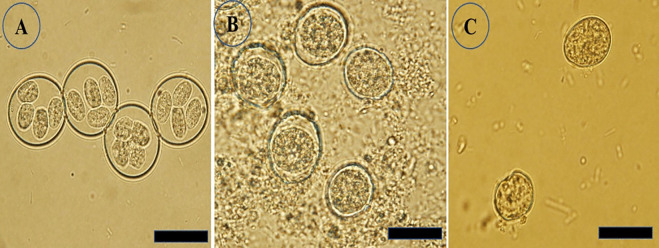
Representative microscopic images of oocyst development showing (A) sporulated oocysts, (B) inhibited sporulation, and (C) destroyed oocysts. Scale bar 20 µL.

The figures indicate that both *Laurus nobilis* and *Cinnamomum verum* extracts exert a clear, concentration‑dependent inhibitory and destructive effect on *Eimeria columbae* oocysts after 96 h in vitro, with higher doses approaching the sporulation‑inhibitory performance of the reference drug amprolium compared with the potassium dichromate control (Fig 7).

**Fig 8:**
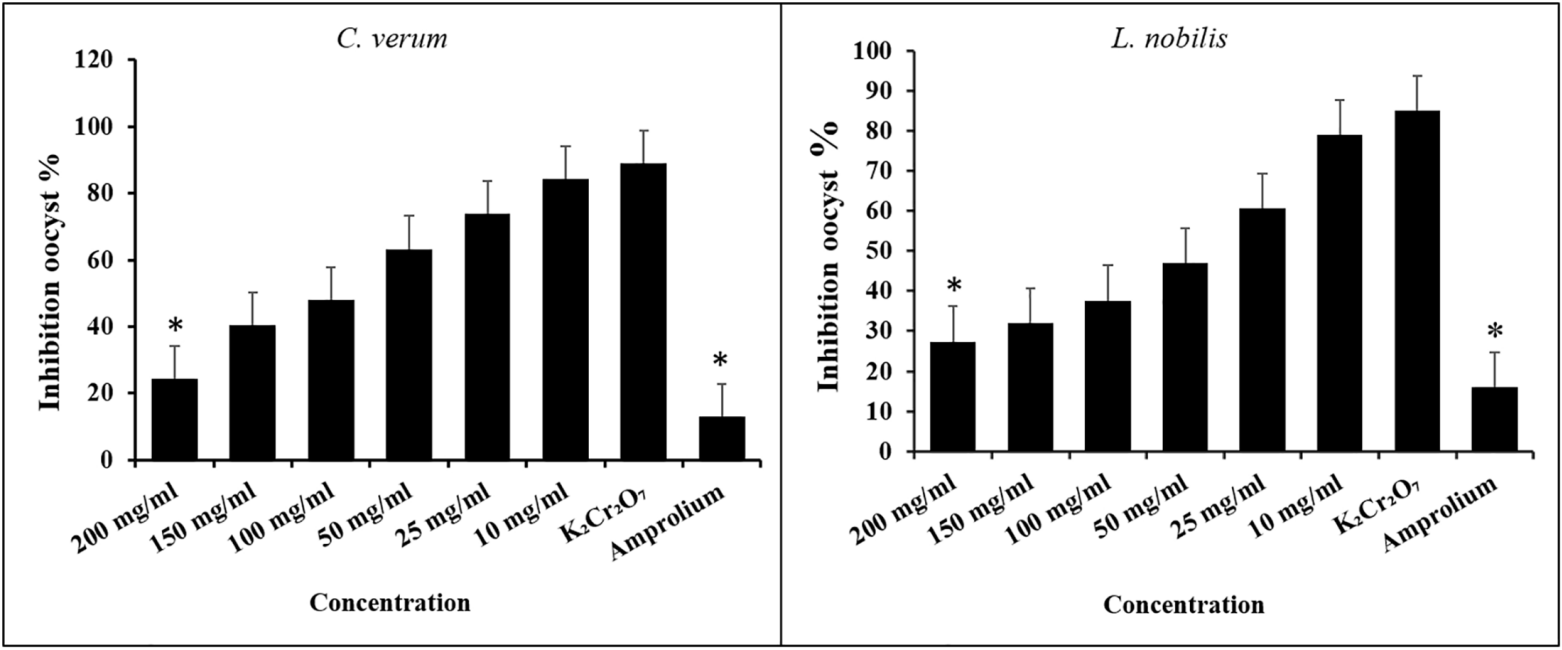
Effects of *C. verum* and *L. nobilis* extract on the inhibition of *E. columbae* oocysts at different concentrations compared to the control (K₂Cr₂O₇) and reference drug (Amprolium), in vitro. * significant differences compared to the control, (*P* < 0.05). n = 3. h, hours.

At 96 h, the figure showed a clear concentration-dependent increase in oocyst inhibition for both *C. verum* and *L. nobilis* extracts in vitro. Inhibition rises progressively from the lower doses to the highest dose, reaching the greatest effect at 200 mg/ml for *C. verum* and *L. nobilis*. 200 mg/ml of extract concentrations produced inhibition: 75.67% at *C. verum* and 72.57% at *L. nobilis*, while the control (K₂Cr₂O₇) showed little or no inhibitory effect. The results indicate that the highest-dose effects and the reference drug effect are significantly different from the control at p < 0.05. C. verum appears to produce slightly stronger inhibition than L. nobilis at comparable concentrations, especially at the upper doses. Both extracts are active, but C. verum seems to reach a higher inhibitory level overall. Amprolium shows a marked inhibitory effect and serves as the positive reference treatment. This result suggests that both plant extracts have anticoccidial activity against E. columbae oocysts and that the effect is dose-dependent. In practical terms, the higher the extract concentration, the more strongly it suppresses oocyst development or viability (Fig 8).

**Fig 9:**
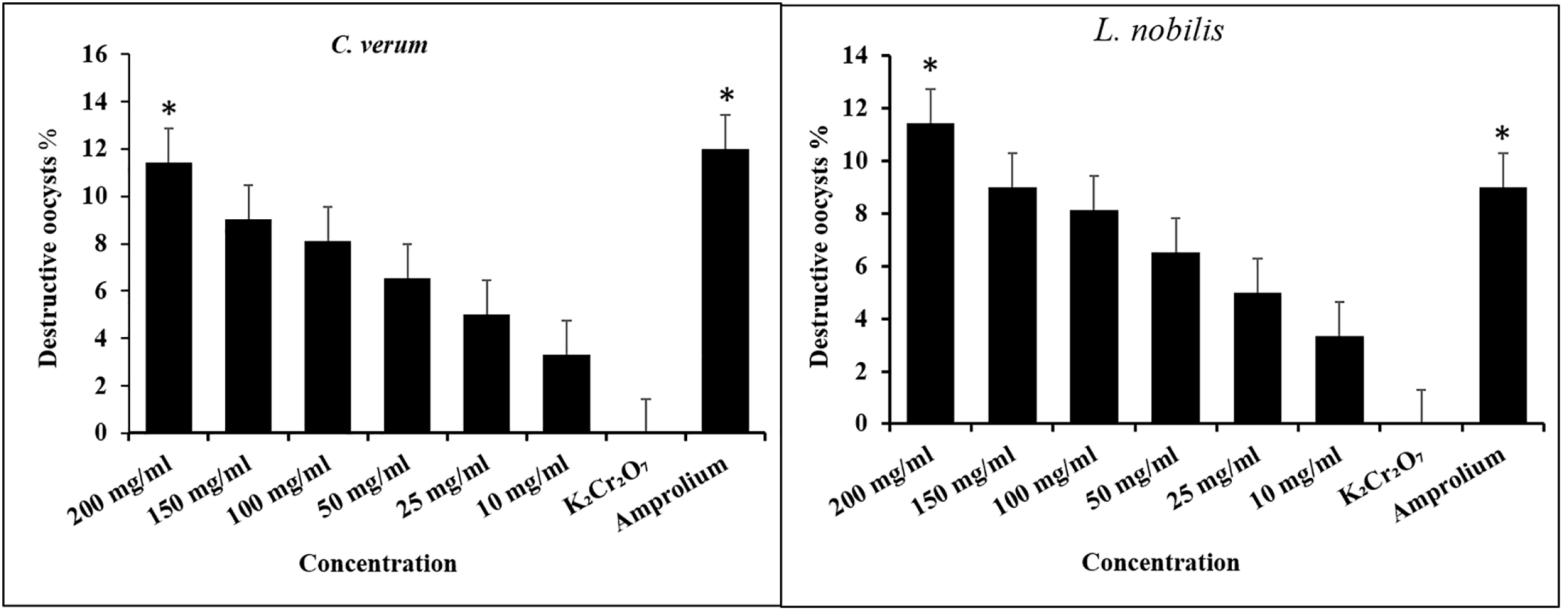
Effects of *C. verum* and *L. nobilis* extract on the destruction of *E. columbae* oocysts at different concentrations compared to the control (K₂Cr₂O₇) and reference drug (Amprolium), in vitro. * significant differences compared to the control, (*P* < 0.05). n = 3. h, hours.

The figure showed a clear dose-dependent destructive effect of both *C. verum* and *L. nobilis* extracts on *E. columbae* oocysts in vitro, with higher concentrations producing greater oocyst destruction. Both extracts performed better than the untreated control (K₂Cr₂O₇), and the asterisk indicates that the observed differences were statistically significant at *P* < 0.05. At 200 mg/ml, both extracts produced the highest oocyst destruction, around 11–12%. As the concentration decreased from 150 to 10 mg/ml, destructive activity gradually declined in both panels. The K₂Cr₂O₇ control shows essentially no destruction, confirming the baseline untreated condition. Amprolium also showed a strong effect, comparable to the highest extract concentrations (Fig 9).

## Discussion

Fourier-transform infrared (FTIR) spectroscopy provided comprehensive spectral fingerprints that confirmed the presence of diverse bioactive metabolites in both *C. verum* bark and *L. nobilis* leaf methanolic extracts. These spectral features align with the known phytochemical profile of cinnamon, particularly the presence of cinnamaldehyde and eugenol, which produce characteristic carbonyl and aromatic signatures [23,24]. Similarly, *L. nobilis* displayed 15 absorption bands, including prominent O-H stretches (3409 cm ⁻ ¹), aliphatic ketones (C = O at 1712 cm ⁻ ¹), and ether linkages (C-O at 1152–1066 cm ⁻ ¹), indicative of terpenoids and polyphenolics [25]. These FTIR results validate the extraction of pharmacologically relevant secondary metabolites, including phenols, flavonoids, tannins, alkaloids, and steroids, which are well-documented for their broad therapeutic potential. Phenolic compounds and flavonoids serve as free radical scavengers, while tannins exhibit protein-binding and antimicrobial properties [26]. The greater spectral complexity in *C. verum* suggests higher metabolite diversity, which correlates with its superior antioxidant performance observed in subsequent assays [27].

Quantitative analysis revealed substantial levels of total phenolic content, tannin, and particularly striking differences in total flavonoid content [28]. These values, derived from robust calibration curves (R² > 0.98 for most standards), demonstrate methanol’s efficacy as a polar solvent for polyphenol recovery [29,30]. The pronounced TFC disparity directly predicts antioxidant capacity differences. However, antioxidant efficacy extends beyond total flavonoid concentration [31,32]. Structural features such as B-ring hydroxylation, 2,3-double bond conjugation, and glycosylation patterns determine individual flavonoid activity. Thus, *L. nobilis* may contain fewer but potentially more bioactive flavonoids, compensating for lower total content through qualitative superiority [33].

The DPPH and ABTS assays provided complementary insights into the extracts’ radical scavenging mechanisms [34]. DPPH (2,2-diphenyl-1-picrylhydrazyl) evaluates hydrogen atom transfer (HAT) capacity through decolorization at 517 nm, while ABTS (2,2’-azino-bis (3-ethylbenzothiazoline-6-sulfonic acid)) assesses both HAT and single electron transfer (SET) via absorbance reduction at 734 nm. This dual-assay approach captures hydrophilic and lipophilic radical interactions comprehensively [35, 36].

Both extracts exhibited concentration-dependent scavenging, with % inhibition increasing linearly from ~20% at 12.5 µg/mL to >90% at 150 µg/mL. *C. verum* achieved significantly lower IC₅₀ values compared to *L. nobilis*, representing 29–58% greater potency (Gulcin et al., 2019). These IC₅₀ values rival synthetic antioxidants and surpass many commercial herbal extracts, positioning both species as viable natural alternatives [37,38].

*L. nobilis* exhibits more variable antioxidant performance (IC₅₀ 15–60 µg/mL), reflecting genetic and environmental influences on polyphenol profiles. The observed moderate activity corresponds to its documented content of 1,8-cineole (20–40%), eugenol, and hydroxycinnamic acids. Notably, laurel’s ABTS scavenging often exceeds DPPH capacity due to hydrophilic polyphenol dominance, consistent with the 1.5 × IC₅₀ disparity observed here [39].

The observed inhibition of *E, columbae* oocyst sporulation by *C. verum* and *L. nobilis* is consistent with the growing body of evidence supporting botanical extracts as potential anticoccidial agents in avian species [12]. Recent studies have shown that methanolic Commiphora myrrha resin extract alleviated oxidative stress, inflammatory injury, and related intestinal damage in pigeons infected with *Eimeria labbeana*-like organisms, suggesting that plant-derived compounds may help mitigate coccidia pathology [40]. In addition, in vitro work on *Calotropis procera* demonstrated inhibitory effects on Eimeria oocyst development, further supporting the notion that plant metabolites can interfere with sporulation and parasite viability [41].

Taken together, the time‑course and dose–response data support that *L. nobilis* and *C. verum* extracts exert potent, sustained anticoccidial effects against *E. columbae* oocysts by hindering or arresting sporulation rather than merely delaying it [42]. The progressive divergence between treated groups and the K₂Cr₂O₇ control with time suggests cumulative damage or persistent metabolic disruption in the sporty, likely mediated by phenolic and terpenoid components that compromise membrane integrity, disturb redox homeostasis, and impair mitochondrial function [43]. Although direct reports on *E. columbae*-specific plant extract studies remain limited, our findings extend this evidence by showing that *C. verum* and *L. nobilis* methanolic extracts possess promising anticoccidial potential under in vitro conditions. These findings highlight the potential of both extracts as promising natural candidates for coccidia control, particularly in strategies aiming to reduce environmental oocyst infectivity and reliance on conventional chemotherapeutics.

## Conclusions

Phytochemical study showed high quantities of phenols, flavonoids, and tannins in *C. verum* and *L. nobilis* methanolic extracts, indicating antioxidant and therapeutic potential. New FTIR characterisation of Saudi-market sources’ phenolic characteristics gives insights for local applications and conservation. Both extracts suppressed *E. columbae* oocyst sporulation in vitro, suggesting coccidial control. However, GC-MS/LC-MS should isolate active chemicals and demonstrate efficacy in vivo.

### Limitations and future plans

*Cinnamomum verum* and *Laurus nobilis* methanolic extracts showed promising antioxidant and in vitro anticoccidial action against *Eimeria columbae* oocysts, although there are significant limitations. The anticoccidial effects were evaluated only in vitro, preventing direct extrapolation to in vivo efficacy, host safety, or field conditions. The analysis was limited to crude methanolic extracts and FTIR functional-group profiling without bioactive compound identification or quantification. Plant material variation (geography, season) and host parameters (toxicity, bioavailability) were ignored. Future research should utilize advanced analytical techniques (e.g., GC-MS, LC-MS) to identify active constituents, perform in vivo trials in pigeons to evaluate therapeutic efficacy, safety, and performance effects, and investigate extract fractionation, dose optimization, and synergistic combinations to improve practical applicability as natural anticoccidial feed additives.
